# Developing the theoretical model of Chinese physical education teachers’ health communication competence: based on grounded theory

**DOI:** 10.3389/fpubh.2023.1233738

**Published:** 2023-12-19

**Authors:** Lilin Chen, Yue Xu, Fangfei Li, Mingzhu Sun, Zhihua Yin, Zhen Guo, Bo Liu

**Affiliations:** ^1^College of Physical Education and Health, East China Normal University, Shanghai, China; ^2^Department of Physical Education and Sport Sciences, University of Limerick, Limerick, Ireland; ^3^Department of Physical Education and Military Training, Zhejiang A&F University, Hangzhou, China; ^4^Department of Physical Education Teaching, Shanghai University of Engineering Science, Shanghai, China; ^5^Division of Sports Science and Physical Education, Tsinghua University, Beijing, China

**Keywords:** health communication competence, physical education teacher, grounded theory, focus group (FG), model development

## Abstract

**Background:**

Physical education teachers’ health communication competence is a key factor in health promotion. Although health communication is a multidisciplinary field, medical practitioners are the primary focus of health communication research, whereas physical education teachers are marginalized. Therefore, this study proposes a theoretical model of health communication competence for physical education teachers.

**Methods:**

This qualitative research utilized interviews as the primary data collection method. Purposeful sampling was employed to select participants, including university teachers, primary and secondary school teachers, and health education professionals from diverse regions of China. A total of 31 participants were interviewed through two focus groups (*N* = 15) and individual semi-structured interviews (*N* = 16). Grounded theory was used to analyze and code the collected interview materials.

**Results:**

The health communication competence of physical education teachers consisted of three main categories, 10 subcategories, 30 concepts, and 240 statement labels. The three main categories were as follows: (i) foundations of health communication knowledge and skills (this category encompassed three subcategories, namely sport and health knowledge reserve, health beliefs, and health behaviors); (ii) health communication perception competence (this category included two subcategories, namely health risk and crisis perception competence and communication audience perception competence); and (iii) practical competence of health communication (this category consisted of five subcategories, namely language expression competence, organizational and design competence, utilization of new media tools competence, communication content selection and processing competence, and professional skills).

**Conclusion:**

The theoretical model of health communication competence in this study provides a foundation for the involvement of physical education teachers in health communication work. It can serve as a reference for the development of both pre-service health education courses and in-service training systems for physical education teachers. Future research can expand the sample size and geographic coverage to further validate the applicability of the findings. Additionally, investigating the factors influencing the formation of the identified competencies is recommended.

## 1 Introduction

Health communication plays a crucial role in promoting healthy lifestyles and enhancing public health literacy (1–3). Additionally, it serves as a focal point in academic discourse. Although health communication has characteristics that span across various disciplines, research in this field predominantly takes place in hospital settings. Examples include controlling smoking or promoting vaccine literacy (4). The primary objective of these initiatives often relies on medical professionals, such as doctors or nurses (5). China believes that exercise is the best medicine; therefore, physical education has been seen as a potential means of health communication. As for the young generation, social media has become the most popular space for them to acknowledge health information and foster health communication (6). As social media is a space where user-generated content (UGC) is generated, health-related communication is problematic, especially when pseudoscientific or commercial information is prevalent (7). In light of the prevalence of health communication in social media, and the threat of “bad money driving out good money,” accurate scientific health communication has become increasingly important.

As physical health among the youth has declined dramatically in recent years (8–11), current research offers significant guidance regarding health communication in physical education, focusing on curriculum change (12), and the development of scientific standards (13). The reform of physical education curriculum means encouraging students to establish healthy lifestyles and values, which requires physical education teachers not only to have professional knowledge in the field of physical education, but also to have knowledge in health education, healthcare, and other discipline. Therefore, physical education teachers need to have certain interdisciplinary teaching abilities (14). With respect to the development of scientific standards, several policies have been launched, including the “Guidelines on Physical Activity and Sedentary Behavior” from the World Health Organization in 2022 (15), “Guidelines on Physical Activity and Sedentary Behavior for Chinese Residents,” and “Guidelines for the Evaluation of Health Literacy of Chinese Residents” from the Chinese government (16, 17). Regardless of the focus, be it curriculum change or the development of scientific standards, the competence of physical education teachers in health communication always emerges as pivotal. The domain of physical education and health is where a physical education teacher’s expertise, both in terms of knowledge and effective teaching practice, is deeply rooted. In essence, the competence of physical education teachers in health communication stands as a key determinant of the success of these health communication policies.

For the objectives of health communication research, medical professionals, including those such as doctors, nurses, and general practitioners, have been central. Their professional development trajectories and models of health communication competencies have been scrutinized (18). Studies have attempted to identify specific health communication competencies for diverse groups. Coleman et al. (13) employed the Delphi approach to establish a theoretical framework for health literacy practice education among in-service medicine practitioners (19). Timothy (20) conducted a questionnaire survey to assess health communication competencies among preservice health professionals at the graduate level. He found that specific elements, such as media, interaction, and health promotion theories, form the core of health communication competence. Park et al. (21) conducted a quantitative study to explore the ideal health communication competence for in-service health educators. The study identified health theoretical foundation, communication skills, and interdisciplinary abilities as key attributes of health communication competence (22).

Rogers introduced the concept of health communication, which essentially refers to any form of human interaction that pertains to health-related content (23). From Rogers’ definition, it is apparent that health communication should span across more relevant disciplines and groups. However, existing health communication research reflects interdisciplinary studies and is primarily a combination of the fields of medicine and communication. Physical education, actively promoting health, is an essential component of any comprehensive health system. However, there is limited research on physical education teachers and their role in promoting health in existing health communication studies. Within the sphere of health communication concerning physical health issues, physical education teachers are more specialized than healthcare professionals such as doctors and nurses. In China, physical education teachers not only need to acquire knowledge in physical education but also study subjects such as exercise physiology, sports health science, sports anatomy, and sports biochemistry. Given the substantial population of in-service and preservice physical education teachers in the 317 universities offering physical education teacher education courses out of 2,688 universities in mainland China (24), they can potentially reach a broader spectrum of professions and individuals when engaging in health communication.

Despite the abundance of research in the field of health communication conducted from various perspectives and proposed resolutions, the role of physical education teachers remains overlooked. Furthermore, the majority of studies are predominantly macro-level, leaving a gap in translating these findings into practical strategies for unique groups, such as physical education teachers engaging in health communication collaboratively. This void constrains the effectiveness of health communication initiatives. Importantly, questionnaire surveys have dominated health communication competence research. In most cases, questionnaire surveys rely on relatively simple questions, which are unlikely to yield complex information (25), while grounded theory is especially useful when the information to be gathered is varied or complex, or when the study is exploratory (26). Since physical education is a complex social phenomenon (27), grounded theory is widely accepted in the field, such as physical activity (21), physical education courses (28), and teacher’s professional development (29). Therefore, this study aims to address this gap by investigating the health communication competence of physical education teachers using grounded theory. This model seeks to offer practical guidance for physical education teachers involved in health communication and introduce a fresh perspective for research in the field of health communication.

## 2 Materials and methods

The study employed grounded theory, which is widely used in research on physical education teachers (30–32). Grounded theory is an approach that avoids preconceived notions and follows a bottom-up process of theory development, making the theoretical model constructed more objective (33). It is characterized by its focus on practice, reliance on empirical evidence, and foundation in real-world experiences. As a typical qualitative research method, grounded theory not only possesses the holistic characteristics common to all qualitative research methods but also assists researchers in delving deeply into the experiences, beliefs, and emotions of the research subjects (29). This method gradually facilitates a more profound understanding and interpretation. The bottom-up approach to theory generation inherent in grounded theory ensures the thoroughness and authenticity of the constructed theory (34). Therefore, the theoretical models developed through grounded theory are more intricate, offering better practical guidance and making them suitable for constructing theoretical frameworks (26). The reliability and accuracy of the original data have a significant impact on the results of grounded theory. These characteristics are particularly valuable in the current context in which there is a lack of research on the health communication competence of physical education teachers. Grounded theory can help physical education teachers quickly establish a comprehensive awareness of the competence needed for health communication, enabling a rapid immersion into health communication practices.

### 2.1 Participants

The reliability and accuracy of the original data have a significant impact on the results of grounded theory. The requirements of grounded theory for interviewees were reflected in three aspects: (i) the number of participants; (ii) the approach of interviews; (iii) the representativeness of interviewees. For the number of participants, according to Guest et al. (35), 12 interviews were sufficient to reach data saturation. When considering the huge population size in the context of China, additional interviews provided more nuanced insights. Hence, a total of 31 participants were selected by a purposive sampling approach in this study. Specifically, 16 participated in individual interviews and the remaining 15 were divided into two focus groups to gather the data.

The first focus groups were conducted with nine participants, and six participants joined in the second focus group sessions. Based on the limited research available regarding the health communication competence of physical education teachers, the number of interviews provides another reason to construct a theoretical model of the health communication competence among Chinese physical education teachers. For the approach of interview, qualitative research emphasizes in-depth exploration and understanding of participants’ experiences, perspectives, and insights (36). By conducting individual interviews and focus groups in the field of physical education teacher, we can gather a rich and diverse range of data from both individuals and group discussions and interactions that may generate additional insights (37).

The representative questions were mainly based on the results of grounded theory. In order to enhance the scientificity and practicality of the theoretical model constructed by the research institute, this study not only selected primary and secondary school physical education teachers as interviewees, but also selected research scholars in health communication and health communication practitioners (nurses) as interviewees (see Table 1 for details of the participants). The inclusion criteria of the participants included: (i) a minimum of 2 years teaching experience in physical education; or (ii) a minimum of 2 years working experience in health communication as health education professionals. Prior to the interviews, informed consent was obtained, and the interview process was recorded with the participants’ permission.

**Table 1:** Information about the interview participants (*N* = 31).

Interview participants (*N* = 31)		
Teaching experience (years)	0–10	20 (65%)
	10–20	2 (6%)
	20–30	4 (13%)
	30–40	5 (16%)
Degree	College diploma	1 (3%)
	BEd	9 (29%)
	MEd	16 (52%)
	PhD	5 (16%)
Title	Second-level teacher	15 (49%)
	First-level teacher/assistant professor	4 (13%)
	Associate senior teacher/associate chief nurse/associate professor	10 (32%)
	Professor	2 (6%)
Region	Northern China	2 (6%)
	Eastern China	19 (61%)
	Central China	1 (3%)
	Southwest China	6 (20%)
	Northwest China	3 (10%)
Interview method	Individual interview	16 (52%)
	Focus group interview	15 (48%)

The professional titles of Chinese primary and secondary school teachers are divided into five main levels: third-level teachers, second-level teachers, first-level teachers, associate senior teachers, and senior teachers. Professor in university is equal to senior teacher in primary and secondary school. Associate professor is equal to associate senior teacher or associate chief nurse. Assistant professor is equal to a first-level teacher.

### 2.2 Interview

The ethical review was approved by the author’s institution. The interview questions were informed by relevant literature reviews (38–40), as well as the insights that were gathered from three experts before the interviews began. A physical education expert and two experts in the fields of communication and health education were selected to participate in this pilot study. As a result of their expertise, we refined the interview outline to ensure that the interview questions were aligned with the research inquiries that were being addressed in the interview. The study ultimately designed four open-ended questions (see Table 2), which were mainly focused on two aspects: (i) the interviewees’ experiences in health communication and (ii) the dimensions of health communication among physical education teachers. Owing to geographical distances, respecting the subjective preferences of the interviewees, and the lockdown policy related to the COVID-19 pandemic in China, interviews were primarily conducted online.

**Table 2:** Interview questions (*N* = 4).

No.	Interview questions
1	Have you ever engaged in health communication with your family and friends? If so, please provide examples?
2	How do you define the health communication competence of physical education teachers? What aspects are included in this competence?
3	What roles and responsibilities do you think physical education teachers have in health communication? How do they contribute to promote pupils’ healthy behaviors and awareness?
4	What are the main challenges that physical education teachers face in health communication? In which areas do they need further improvement?

Each interview took 45–60 min and adhered to the guidelines established by the interview guidance on sports, exercise, and health (25, 27, 41): (1) the list of questions should be as concise as possible, incorporating probing questions to extract additional information from the participants and delve deeper into their accounts, responses, and stories. Our study’s probing questions were developed based on the interview guidance provided by Clegg and Butryn (19) in the field of physical education (42). For instance, the first interview question in our study is, “Have you ever engaged in health communication with your family and friends? If so, please provide examples.” Following Clegg and Butryn’s guidance, we developed sub-questions such as: how has such engagement with friends/family affected you? What thoughts stood out for you in a special moment during health communication? How do you feel when you imagine engaging in health communication with your family or friends, even if you have not done so yet? (2) Kvale (43, p.103) suggests that the sample size of qualitative research is not one that should be determined at the beginning of a study (44). Additionally, Baker (45) notes the basic rules of how to become a good interviewer in social science: the researcher should be committed at every interview to spend sufficient time to gather enough data. Therefore, the 45–60 min allocated for each interview was determined based on the conversations with interviewees, concluding each session when saturation was achieved. Owing to the dynamic nature of conversations in focus groups and the emergence of unexpected thoughts during group interactions (25), focus group sessions in our study lasted slightly longer than individual interviews.

### 2.3 Data analysis

After the interviews, the audio recordings were first converted into text using Microsoft Word software. The transcriptions were then organized and read through, and irrelevant content was removed. Through both individual interviews and focus groups, the researchers compiled a total of 18 interview transcripts, amounting to 44,736 words. Next, the interview data were analyzed using the three-level coding approach of grounded theory with Nvivo19 software. The coding process involved open coding, axial coding, and selective coding (26). This analysis aimed to construct a theoretical model of health communication competence for physical education teachers in China.

#### 2.3.1 Open coding

Open coding is the process of the initial screening and analysis of the collected interview data in the research. It can be understood as the process of “labeling” the data. During this process, irrelevant content was removed to ensure that the coding was not influenced by subjective understanding or existing research. Relevant statements were analyzed and extracted to form labels and categories for related concepts.

Step 1: Labeling involves the process of transforming interview data into labeled statements, as described in Table 3. The labels in the text follow the naming convention of “EN + label statement,” for example, “E1 You should have healthy behaviors and lifestyle.” A total of 240 labeled statements were generated (see Table 4). The labels indicate that the interviewed teachers and experts primarily focused on several aspects of the components of health communication competence for physical education teachers, such as language expression, knowledge reserves, and the use of information technology devices.
Step 2: Conceptualization. Through comparing, refining, analyzing, and integrating the labeled statements, similar or related contents were merged, further refining the 240 labeled statements generated in the labeling process. In this study, 30 concepts were integrated from the 240 labeled statements. The concepts are presented using the format “DN+ Concept Name.” For example, D1 Health Lifestyle. Detailed examples of the process from labeling to conceptualization can be found in Table 5, and the results of conceptualization are shown in Table 6.
Step 3: Categorization. Categorization was achieved through continuous analysis, comparison, and induction of the 30 concepts generated. From these concepts, categories were extracted, emphasizing the need for high exclusivity among various categories and high representativeness of the concepts within each category. Through this process, a total of 10 categories were identified. The categories are presented using the format “CN + Category Name,” e.g., “C1 Sports and health knowledge reservoir.” Detailed examples of the categorization process can be found in Table 7, and the complete results of categorization are shown in Table 8.

**Table 3:** Examples of the tagging process for the interview data.

Type	Interview material	Labeling
Focus group interview 1	In subjects like emergency safety, preparedness, and risk mitigation, it is necessary for you to be able to guide students on what to do. Therefore, when imparting this knowledge to students, you should possess the necessary knowledge and skills.	E4 Know how to guide students to do so when an emergency situation happens
		E5 You should possess health communication relevant knowledge and skills
Individual interviewee, Individual interview materials 16	When you have a strong grasp of professional knowledge, it becomes easier for others to accept the health information you communicate. It is important to prioritize simplicity and ease of learning when conveying this knowledge. From a wide range of content, you should select the ones that are simple and easy to understand, as this will yield better results. Additionally, it is essential to stay informed about currently popular and trending fitness activities. This knowledge will accelerate the dissemination process because these activities are both easy to learn and engaging, leading to a rapid spread of information.	E108 Possess strong professional knowledge to enhance credibility
		E109 Select simple and easy-to-learn content from a variety of materials
		E110 Know what popular and trending fitness activities are currently

**Table 4:** Labeling results.

Tagged labels	Tagged labels
E1: It requires a healthy behavior and lifestyle	E121: You need to know the mainstream communication platforms
E2: The basic knowledge is important	E122: You should understand how to train overall physical fitness
E3: Skills are also important	E123: Can design various exercise methods
E4: Know how to guide students to do so when an emergency situation happens	E124: Can coordinate his spare time or his time after work
E5: You should possess health communication relevant knowledge and skills	E125: It requires knowledge and ability to prevent some of the most basic and common diseases
E6: Has a relatively rich understanding of health education and health communication	E126: The knowledge of sports and health in books is a fundamental requirement for communication

For the specific date, see Appendix Table A1.

**Table 5:** Example of the process from raw materials to conceptualization.

Conceptualization	Labeling	Interview material
D6: Can use the internet for communication	E18: Should catch the fast train of epoch development	Contemporary physical education teachers should hop on the fast-moving train of technological advancements to effectively disseminate health knowledge. It is crucial to leverage popular short video platforms like TikTok and Kwai to ensure the rapid spread of our knowledge. This requires physical education teachers to have the ability to utilize information technology and to filter through various sources of information.
	E19: Should be able to grasp the peak period of short video platforms such as TikTok and Kwai	
	E20: Have the ability to apply information technology	
D8: Having passion for health communication	E17: Should have a pure intention of helping people engage in healthy behavior	Currently, much of the available health information is driven by profit and heavily influenced by commercial interests. Therefore, it is important for physical education teachers to maintain pure intentions and prioritize public welfare when engaging in health communication.

**Table 6:** Conceptualization results of 240 label statements.

Conceptualization numbers	Tagged labels
D1: Having a healthy lifestyle	E1, E43, E53, E102
D2: Possessing relevant knowledge of sports and health	E2, E5, E6, E7, E8, E11, E35, E49, E58, E73, E79, E90, E91, E108, E114, E122, E125, E126, E128, E132, E167, E178, E179, E186, E187, E197, E198, E206, E207
D3: Having relevant communication skills	E3, E34, E54, E55, E97, E100, E103, E168, E177
D4: Leading by example	E12, E13, E14, E44, E84, E85, E156, E160, E181, E193, E195, E238

For the specific date, see Appendix Table A2.

**Table 7:** Example of the categorization process of raw materials.

Categorization	Conceptualization	Labeling	Interview material
C6: Language expression competence	D16: Possessing good language expression and organizational skills	E80: Making others understand is essential	I believe there is a need for effective verbal expression because, in reality, you have to communicate with others. Sometimes, we have to convey something to others and ensure that they understand it. It all comes down to how you organize and articulate your language. You may possess knowledge yourself, but when it comes to conveying it to others, it may not be as straightforward.
		E81: The organization and expression of language are crucial	
	D24: Having the ability to facilitate communication and collaboration	E104: Expand beyond the school environment and have the ability to communicate and collaborate	If we aim to expand beyond the school environment, it is essential for physical education teachers to engage in communication and collaboration with the community.

**Table 8:** Categorization results of 30 concepts.

Categorizations	Conceptualization	Label numbers
C1: Sports and health knowledge reserve	D2: Possessing relevant knowledge of sports and health	29
	D18: Demonstrating professionalism when communicating	11
C2: Health beliefs	D8: Having passion for health communication	8
	D26: Being adaptable to different roles	4
C3: Healthy behavior	D1: Having a healthy lifestyle D4: Leading by example	4
		12
C4: Health risk and crisis perception competence	D5: Seizing special opportunities for health communication D27: Can seize current hot topics	9
		5

For the specific date, see Appendix Table A3.

#### 2.3.2 Axial coding

Axial coding is the second level of coding in the grounded theory three-level coding process. It builds upon the concepts and categories formed in the initial coding stage. In axial coding, the focus of our study was on analyzing the causal relationships, interactive strategies, and phenomena among the categories obtained from the open coding process. In this study, three main categories, denoted as BN (Basic Categories) followed by their names, were identified. The three main categories were as follows: B1: Foundation of Health Communication Knowledge and Skills, B2: Health Communication Perception Competence, and B3: Practical Competence of Health Communication (refer to Table 9). The category “B1 Foundation of Health Communication Knowledge and Skills” includes three subcategories: C1: Sport and health Knowledge Reserve, C2: Health Beliefs, and C3: Health Behaviors. The category “B2: Health Communication Perception Competence” consists of two subcategories: C4: Health Risk and Crisis Perception Competence and C5: Communication Audience Perception Competence. The category “B3: Practical Competence of Health Communication” encompasses five subcategories: C6: Language Expression Competence, C7: Organizational and Design Competence, C8: Utilization of New Media Tools Competence, C9: Communication Content Selection and Processing Competence, and C10: Professional Skills. These main categories and subcategories represent a comprehensive and condensed theoretical model of health communication competence for physical education teachers, derived from the in-depth understanding of the research participants.

**Table 9:** Results of the main axis coding for 10 categories.

Subcategories	Subsidiary categories
B1: Foundation of health communication knowledge and skills	C1: Sports and health knowledge reserve
	C2: Health beliefs
	C3: Healthy behavior
B2: Health communication perception competence	C4: Health risk and crisis perception competence
	C5: Communication audience perception competence
B3: Practical competence of health communication	C6: Language expression competence
	C7: Organizational and design competence
	C8: Utilization of new media tools competence
	C9: Communication content selection and processing competence
	C10: Professional skills

#### 2.3.3 Selective coding

**Figure 1:**
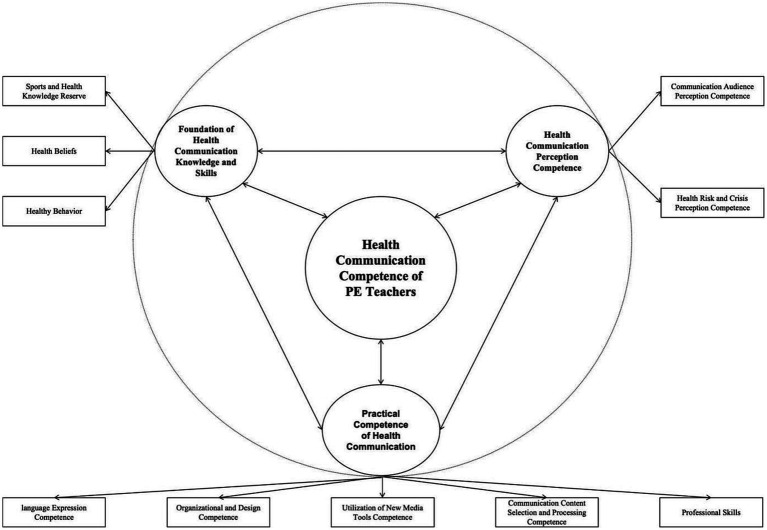
The health communication competence of physical education teachers.

Selective coding is the final process in constructing the grounded theory three-level coding. Its main purpose is to distill the “core concept” that can encompass all the concepts (26). This core concept must have a unifying nature and be able to encompass the majority of the research findings. Through in-depth analysis of the 10 subcategories and three main categories formed, combined with the concepts and labels identified in the previous coding process, and considering the original interview content, the core category that emerged, encompassing all other categories, was “Health Communication Competence of Physical Education Teachers.” This study followed the three-level coding process in grounded theory, progressing through the processes of labeling, conceptualization, and categorization in the first-level coding, main categorization in the second-level coding, and core categorization in the third-level coding. The three-level coding resulted in the theoretical model of health communication competence for physical education teachers, which includes three main categories, 10 subcategories, 30 concepts, and 240 statement labels (see Figure 1; Appendix A).

### 2.4 Trustworthiness

To ensure the credibility of the components of physical education teachers’ health communication competence, a coding process was conducted first for individual interviews, with the data from two focus group coded after the completion of the individual interviews. This process did not result in the emergence of new concepts or categories. Additionally, three sets of materials were reserved in advance during the coding process of individual interviews. After completing the coding, these reserved materials were coded as well, but no new concepts or categories were identified. Finally, the data collected from the individual interviews were coded, followed by the selection of one interviewee for further interview coding. However, no new concepts or categories were discovered. Therefore, the constructed framework for physical education teachers’ health communication competence in this study was considered saturated and aligned with the research requirements.

## 3 Results

Based on the three-level coding process of grounded theory, it can be observed that the health communication competence of physical education teachers can be primarily described through the overall framework of Basic Health Communication Knowledge and Skills, Perception of Health Communication, and Practical Ability of Health Communication. The combination of these main categories and subcategories provides a comprehensive depiction of the theoretical model of health communication competence for physical education teachers.

### 3.1 The foundation of health communication knowledge and skills

From the constructed categories, it is evident that physical education teachers need to possess a strong reservoir of physical education and health knowledge to communicate health effectively to promote healthy behaviors and awareness in pupils. The need for physical education and health knowledge was also mentioned frequently by participants.

#### 3.1.1 Sports and health knowledge reserve

When engaging in health communication, physical education teachers primarily focus on content related to physical education and health. Health knowledge, particularly physical education health knowledge, is highly time-sensitive, and topics related to health also entail a certain level of professional expertise. Therefore, physical education teachers can only carry out health communication successfully when they have acquired a certain theoretical foundation. This point is raised in response to the prevailing issue of “pseudo-experts” in the field of health communication.

“*To promote physical education and health, it is essential for us to possess relevant knowledge in this field. Teachers must understand which exercise can lead to specific outcomes of health promotion. Therefore, equipping professional knowledge in physical education and health is crucial for physical education teachers*” (Individual interviewee, Individual interview materials 03).

#### 3.1.2 Health beliefs

Health beliefs reflect the long-term and public welfare nature of health promotion work. The effectiveness of health communication may not be immediately apparent, which requires physical education teachers to have firm beliefs. Physical education teachers should have confidence and enthusiasm in their health communication work. The proposal of this ability also reflects the common profit-seeking problem in the field of health communication. Health communication should primarily serve the public interest. However, due to the lack of regulation in this field, many health communication entities prioritize profitability, leading to a certain consumer-induced tendency in the dissemination of health information.

“*A physical education teacher needs to persevere in their efforts because the effects of health communication may not be immediate. Therefore, they must have faith in the value of the health communication work they are engaged in*” (Individual interviewee, Individual interview materials 04).

“*There is currently a strong commercial influence in the field of health communication. Many health-related contents disseminated through the internet have some commercial purposes. For example, during the process of health communication, there are instances where products are inadvertently promoted. Therefore, for physical education teachers to effectively engage in health communication, they need to first recognize the underlying public welfare aspect of this matter and possess a certain level of belief in it*” (Individual interviewee, Individual interview materials 02).

#### 3.1.3 Health behavior

Health behavior, as a capability, reflects the current trend in the Chinese health communication field characterized by an “emphasis on theory, neglect of practice.” While health education professionals, such as doctors and communication scholars, possess substantial health knowledge, they also engage in sedentary behaviors. The most significant deficiency in the field of Chinese health communication is a communicative agent that not only possesses theoretical knowledge but also serves as a behavioral model. Overemphasizing the transmission of theoretical knowledge can lead to diminishing returns in the effectiveness of health communication in practice. Therefore, physical education teachers themselves also need to maintain healthy behavior in order to become role models for students and effectively ensure that complex health knowledge is put into practice.

“*When engaging in health communication, we focus on not only slogans and knowledge dissemination but also providing practical examples. For instance, an* [*sic*] *user called Genghong Liu in Douyin (a social media platform similar as TikTok), who guiding other online users for physical fitness training by live streaming [sic]. During his live streaming, more than 100,000 online users participated in exercise. This type of online physical fitness course embraced by online users was more effective than delivering health education classes or physical education lessons on campus*” (Individual interviewee, Individual interview materials 10).

### 3.2 Practical competence of health communication

Health communication work possesses a certain level of complexity, requiring physical education teachers to have relatable abilities to coordinate various influential factors to achieve maximum effectiveness.

#### 3.2.1 Language expression competence

Language expression skills were mentioned by all participants. Strong language expression skill is crucial for physical education teachers to effectively delivery health communication work. With the development of information technology, physical education teachers need to consider how to make complex sports and health knowledge more appealing for pupils when using various social media platforms for communication. Excessive specialization in health knowledge can limit the scope of communication. Therefore, overcoming the issue of diverse cultural literacy among the audience during the communication process is essential, and the role of language expression skills becomes particularly significant. Additionally, the ability to interact with the audience during communication places higher demands on language expression skills. This ability also allows them to establish a personal brand and form the basis of a communication community.

“*I think language expression is necessary because when you want to communicate with pupils, you need to make sure they can understand what you teach. It’s essentially a form of language expression. You may have the health knowledge yourself, but when you explain it to pupils, it may be misunderstood by others. So, I believe that art of language matters, namely physical education teachers need to know how to express ourselves*” (Individual interviewee, Individual interview materials 09).

“*Firstly as a physical education teacher, you definitely need the ability to express yourself through language during teaching, specifically the ability to organize your health knowledge. It’s similar to how an expert delivers a lecture in any other field. An expert must possess language expression skill to convey their knowledge in the simplest and most understandable way possible*” (Focus Group Interview Material 01).

#### 3.2.2 Organization and design competence

The organizational design competence suggests that the requirements of diverse communication channels in current health promotion. Whether the health communication among physical education teachers is online or offline, teachers need to possess skills in organizing activities, particularly in coordinating time and space. Given the unevenly developed fitness-related infrastructure across the different regions of China, organizing health communication activities poses challenges for physical education teachers. Additionally, the ability to design effective activities is crucial as it determines the quality of physical education teachers’ health communication work and plays a significant role in maintaining the stability of the communication community.

“*In the context of health communication, one approach is to design practical fitness tests or health-related exercises such as sports injury prevention. Students can absorb health knowledge easily and authentically by participating in health-related activities. Another approach is to implement health-related tests or competitions focusing on enhancing health levels instead of judgment. By receiving feedback from such a test or competition, students can get chance to understand how to enhance and use the health knowledge they learned from physical education teachers. These initiatives require physical education teachers to possess adequate abilities in activity organization and design*” (Focus Group Interview Material 02).

#### 3.2.3 Utilization of social media competence

With the development of technology, the context of health communication has shifted to social media (46). In China, WeChat and Douyin are the primary social media for health-related information dissemination, especially among the young generation. This transformation is mainly driven by the convenience of accessing health information through social media, which has broken the monopoly of traditional media in the dissemination of health information. Therefore, owing to an increasing number of pupils learning health-related knowledge from social media, physical education teachers need to learn the wisdom of social media to support pupils in the new era. Moreover, social media facilitate the formation of virtual communication communities, which potentially provide opportunities for emotional support between teachers and pupils. In turn, it would benefit the health communication for physical education teachers.

“*There are two key requirements for creating effective health-related videos through social media. First, health-related content expression ability is essential, especially physical education and health knowledge required more authentic experience than other subjects such as math and English [sic]. Second, proficiency in using social media, such as video editing, incorporating* var*ious materials. These abilities are crucial for conducting health communication in any social media platforms [sic]*” (Individual interviewee, Individual interview materials 13).

#### 3.2.4 Communication content selection and processing competence

The ability to select and process communication content for health promotion requires physical education teachers to choose appropriate content based on their own pupils’ needs. Unfortunately, the content of the health communication delivered by physical education teachers will more likely promote public health literacy and health awareness rather than specifically target their own pupils. Thus, the main form of health communication should be refined by considering pupils’ needs instead of the general public.

“*To establish a comprehensive health system, it is essential to focus on specific aspects rather than being too broad. Physical health encompasses a wide range of topics, and there are numerous activities that can be included. For instance, exercises like Tabata involve* var*ious physical movements, and many other fitness exercises can be incorporated as well. While the exercise for adults might not acceptable for students, so it is important to choose a specific project and refine it to cater to the target audience [sic]. By focusing on a particular activity and ensuring its precision and effectiveness, a more targeted approach can be achieved in health communication*” (Individual interviewee, Individual interview materials 01).

#### 3.2.5 Professional skills

Professional skills in health communication refer to two aspects. First, it pertains to the physical abilities and demonstration skills of physical education teachers themselves. Owing to the specificity of professional identity among physical education teachers, the content of health communication taught by physical education teachers focused on sports and physical training. Second, professional skills in health communication implicates skills in sports injuries, such as injury prevention, the handling of emergency sports injuries, and sports rehabilitation. In conclusion, subject matters are the prominent factors that distinguish the core duty of physical education teachers compared with those of other health communication practitioners.

“*For a physical education teacher, it is essential to have proficient physical abilities and at least be capable of demonstrating standard movements. Additionally, teach health communication for physical education teachers means teach sports injury prevention and possess knowledge of sports rehabilitation methods [sic]. Therefore, physical education teachers need to acquire these relevant professional skills for effective health communication*” (Individual interviewee, Individual interview materials 11).

### 3.3 Health communication perception competence

The perception competence of health communication plays a vital role in enhancing the breadth and depth of health communication for physical education teachers within the overall health communication competency model.

#### 3.3.1 Health risk and crisis perception competence

When it comes to health communication, most of the participants mentioned competence related to risk and crisis perception. Participants suggested that the aim of health communication is to enhance the effectiveness of health communication by improving cognition at the behavioral level and seizing specific opportunities for pupils. Theoretically, as for physical education teachers, the purpose of health communication is to influence behavior through cognitive changes, which is similar to the fundamental “knowledge-attitude-behavior (KAP)” model in the field of health communication.

“*For example, if one of students gets injured during course, the physical education teacher should perceive this challenging situation as an opportunity for teaching pupils about how to deal with such injuries [sic]. It includes not only teaching students how to handle emergency sports injuries in class, but also educating them on injury prevention and self-evaluating physical condition during after-class physical activities. This situation puts physical education teachers’ perception ability of health crises to the test*” (Focus Group Interview Material 01).

#### 3.3.2 Communication audience perception competence

The concept of audience perception competence in health communication arose from the call for professional development trends in the competency of physical education teachers in health communication. It also aligns with the capacity for selecting and processing communication content. In other words, health communication content should strive for refinement. The need for refinement stems from the fact that health communication often targets diverse audiences, which vary in terms of age, gender, physical condition, and even job type. For example, office workers are more likely to have sedentary behaviors, whereas physical workers or teenagers are less likely to have sedentary behaviors. Therefore, physical education teachers need to understand the specific needs of their students, whether they are university students, high school students, or primary students.

“*To achieve greater scientific accuracy and appropriateness, it is necessary to identify different population groups and age ranges, rather than relying on intuition or general statements. There should be established standards to guide the standardization and meticulousness of health knowledge in this regard. I believe it is essential to pay close attention to the standardization and specificity of health knowledge. Even if individuals have some understanding, it cannot be claimed to be 100% accurate. Therefore, targeted health knowledge is more scientific and accurate*” (Individual interviewee, Individual interview materials 04).

## 4 Discussion

### 4.1 Interrelationships among all subcompetences in health communication competence

The objective of this study was to examine the health communication competence of physical education teachers through the application of grounded theory. The goal was to provide practical guidance for physical education teachers engaged in health communication and bring a new perspective to research in the field of health communication. Based on the constructed health communication competence for physical education teachers in our study, the health communication competence of physical education teachers can be categorized into three main sub-competences: health communication knowledge and skills, health communication perception competence, and practical competence of health communication.

These subcompetences are interconnected and mutually influential. For instance, physical education teachers must possess sufficient health communication knowledge to develop a strong belief in health communication and maintain a sustainable healthy lifestyle (47). Similar to the health communication competence explored by the objectives of medicine practitioners from Clifford (2013) (19) and Park (21), practical competence in health communication is crucial for teachers to effectively apply their health communication knowledge in practice, guiding their behaviors when engaging in health communication activities.

This becomes particularly relevant in the context of new media, as physical education teachers need to understand how to utilize social media platforms to disseminate appropriate health communication information (32). Furthermore, health communication perception competence plays a vital role. Only when physical education teachers recognize the value of maintaining good health and perceive the risks associated with an unhealthy lifestyle, will they be more motivated to work in health communication. Additionally, they will be inclined to learn more about the specific health communication needs of different individuals and show enthusiasm for participating in or establishing health communication communities.

### 4.2 Professional identity as opinion leaders and communication agents in physical fitness

Among all health professionals, physical education teachers have different responsibilities than doctors or researchers in the field of communication. While doctors focus on areas such as pharmacology, disease diagnosis and management, clinical skills, and patient assessment, researchers in health communication specialize in competence related to persuasion and negotiation and verbal and non-verbal communication (18). Physical education teachers, on the other hand, have unique responsibilities that require them to learn from doctors and researchers in the field of communication but with a primary emphasis on physical fitness (43, 48–50).

Their professional identity is reflected in categories such as C1 sports health knowledge reserve, C3 health behavior, and C10 professional skills, which highlight their expertise in sports and physical health. Chinese physical education teachers are required to acquire theoretical knowledge of physical health, exercise physiology, and other exercise-related theories. When they become physical education teachers in schools, colleges, or universities, they also need to systematically learn health promotion practices and training. Therefore, physical education teachers are well-positioned to take on leadership roles and act as agents in the realm of health communication, particularly in promoting physical fitness. It is important to note that the specialization of physical education emphasizes the significance of health practices rather than solely delivering health knowledge. This highlights the need to address the current issue of overemphasizing theory and undervaluing practical aspects in health communication. This change cannot be easily achieved by other health professionals, such as doctors or researchers, alone.

Consequently, it is crucial for physical education teachers to actively engage in health communication work and acquire the necessary competence to effectively deliver their expertise and collaborate with other health communication professionals to maximize outcomes. The theoretical model of physical education teachers’ health communication competence underscores their professional identity, positioning them as opinion leaders or agents in the field of sports health and fitness communication. They commit to bridging the gap between theory and practice in health communication.

### 4.3 Physical education teachers’ health communication competence guides professionalization

The study on physical education teachers’ health communication competences highlights the necessity of acquiring sufficient physical education and health knowledge (51). In today’s context of social media, in which anyone can freely voice their opinions, health-related information often becomes distorted for profit-seeking purposes (6). Physical education teachers should take an active role in navigating digital health communication, particularly in promoting physical health. However, the integration of health into physical education is still in its early stages, and it remains voluntary rather than compulsory for physical education teachers.

This implies that they need to make efforts in their professional development journey toward health and commit to behaving like health professionals (52). The professional development path for health communication practice should encompass areas such as sports health knowledge, sports injury prevention, sports rehabilitation, physical training, and fun fitness training (12). Additionally, physical education teachers need to recognize that the audience for health communication extends beyond just students in school (53). Therefore, the professional development path for health communication practice also emphasizes the active understanding of different target groups and acknowledging their specific needs in health promotion. Furthermore, in the context of new media, health communication takes on a different form than with offline contexts. Physical education teachers should embrace new media platforms, such as TikTok, Kuaishou, and WeChat, which are popular online spaces for the public (43, 54, 55). These platforms can serve as tools for health communication and provide opportunities for physical education teachers to learn from other health communication professionals.

Although health communication for physical education teachers predominantly occurs in the digital space, it is important to stress the combination of online and offline approaches (56). Physical education teachers’ health communication work should not be limited to knowledge alone but also involve practical implementation. This could include offline activities such as disseminating sports rehabilitation exercises and promoting healthy physical training methods. In conclusion, the theoretical model of physical education teachers’ health communication competence suggests a clear professional development path that includes acquiring health-related knowledge, properly utilizing new media platforms, and understanding the specific needs of diverse target audiences.

### 4.4 The cross-disciplinary integration trend in health communication competence

The model of physical education teachers’ health communication competence constructed in our study encompassed a range of subcompetences that extended beyond the field of physical education. This implies that physical education teachers need to step out of their comfort zone and engage in broad learning (57). In today’s context, public health needs have become increasingly individualized and complex (58), and relying solely on a single discipline may no longer be sufficient (59). The theoretical model of health communication competence clearly indicates that physical education teachers should seek knowledge and insights from professionals in medicine, communication, and even computer science.

Embracing cross-disciplinary approaches poses a challenge for physical education teachers in their health communication work. However, it also serves as a motivation for them to broaden their horizons and seek professional and emotional support from a larger community of health communication professionals beyond the realm of physical education. In fact, cross-disciplinary collaboration has already demonstrated its significance in the field of medicine (60, 61). For instance, exercise health has made notable contributions to post-operative rehabilitation and the prevention of chronic diseases (62). Similarly, the theories of communication can significantly enhance the effectiveness of physical education teachers in flexibly disseminating health knowledge, whether it be in community settings or on a one-to-one basis. Interpersonal and group communication knowledge from communication studies can prove particularly valuable in this regard. Furthermore, computer science plays a crucial role for physical education teachers, as they primarily use digital spaces for health communication (9). Through big data analysis, they can identify the diverse health needs of online users, enabling them to better understand their target audiences and pinpoint the most critical needs among different audience groups.

In summary, the theoretical model of physical education teachers’ health communication competence highlights the importance of embracing cross-disciplinary learning. Physical education teachers not only need to understand their expertise in sports but also expand their knowledge by seeking insights from medicine, communication, and computer science. This approach allows them to effectively address the increasingly individualized and complex public health needs. By broadening their horizons and engaging with a larger community of health communication professionals, physical education teachers can enhance their professional capabilities and provide valuable support beyond the scope of physical education alone.

### 4.5 Limitations and future research

Although the competences of physical education teachers exhibit social and cultural variations, it is important to acknowledge that significant cultural differences exist among different religions in China (63). However, a notable aspect to acknowledge is the study’s sample size, which, although suitable for exploratory research, may not be sufficiently large to comprehensively represent all regions. This limitation poses potential challenges in generalizing the findings to the entire population of physical education teachers across the country or worldwide. To address this limitation and enhance the practicality and scientific validity of the theoretical model, future research endeavors should prioritize expanding the sample size. By including a more diverse and extensive representation of regions, researchers can capture the nuanced variations in cultural and social contexts, making the theoretical model more applicable and applicable across a broader spectrum of other populations or ethnicities.

Furthermore, future research can delve deeper into the various factors influencing the formation process of physical education teachers’ health communication competence, building upon the existing foundation. One avenue worth exploring is the role of social media competence among physical education teachers. This entails a detailed examination of the current state of teachers’ social media skills and their proficiency in conveying health communication knowledge through digital platforms. The research could involve a nuanced analysis of teachers’ social media repertoires, investigating the range of platforms they use and how adeptly they navigate these channels to effectively communicate health-related information. This exploration would not only contribute valuable insights into the proficiency levels of physical education teachers but also provide a deeper understanding of the strategies and approaches they employ to engage with diverse audiences on multiple media platforms.

Moreover, we acknowledge that qualitative research can provide deeper insights into perceptions, attitudes, and beliefs in research on health communication, but our experience has indicated that analyzing qualitative data from semi-structured interviews presents greater challenges than analyzing qualitative data from structured interviews. The transcription of focus group data is particularly challenging due to issues such as voice overlapping and simultaneous speech, which requires approximately 6 h to complete. Additionally, there was a lack of diversity in the conversation because of some individuals dominating it. To enhance data quality, future research should select a location that is noise-free if interviews are to be conducted. To achieve high quality qualitative data, interviewers must be proficient in asking probing questions, possess excellent organizational skills, and be able to guide interviewees toward the research questions politely without causing unwanted topic deviations.

Lastly, it would be valuable for future studies to extend their focus to other professionals involved in health communication, such as doctors, nurses, and health education teachers. Exploring the theoretical model of their health communication competence would provide insights into the unique competences and skills required in their respective fields. This comparative analysis would contribute to a comprehensive understanding of health communication competence across various professional domains.

## 5 Conclusion

The research formed the constitution dimension of health communication competence of physical education teachers through three-level coding guided by the grounded theory approach. It resulted in three main categories: health knowledge and skill, health communication perception competence, and practical competence of health communication. The health communication knowledge and skills included three subcategories: sports and health knowledge reserve, health belief, and health behavior. Health communication perception competence included health risk and crisis perception competence and communication audience perception competence. The practical competence of health communication included five sub categories: language expression competence, organization and design competence, utilization of new media tools competence, communication content selection and processing competence, and professional skills. The three main categories and 10 subcategories were derived from 30 concepts and 240 labels from individual interviews and focus groups. Finally, through theoretical saturation testing, it was proven that the theoretical model of Chinese physical education teachers’ health communication competence has good reliability and validity.

## Data availability statement

The original contributions presented in the study are included in the article/Supplementary material, further inquiries can be directed to the corresponding authors.

## Ethics statement

The studies involving humans were approved by College of Physical Education and Health, East China Normal University, Shanghai, China. The studies were conducted in accordance with the local legislation and institutional requirements. The participants provided their written informed consent to participate in this study. Written informed consent was obtained from the individual(s) for the publication of any potentially identifiable images or data included in this article.

## Author contributions

LC and YX: conceptualization and writing—original draft preparation. ZY, ZG, and BL: methodology and funding acquisition. LC: software. LC, YX, and MS: formal analysis. LC, YX, and FL: writing—review and editing. All authors contributed to the article and approved the submitted version.
