# Supplementary material for: Developing the theoretical model of Chinese physical education teachers’ health communication competence: based on grounded theory
Source: Front Public Health. 2023 Dec 19;11:1233738. doi: 10.3389/fpubh.2023.1233738 (PMC10758496; doi:10.3389/fpubh.2023.1233738)
Supplement: Supplementary file 1 [file Table_1.docx]

**Appendix A**

**Table A1.** Labeling results

| **Tagged labels** | **Tagged labels** |
| --- | --- |
| E1 It requires healthy behaviors and a healthy lifestyle | E121 You need to know the mainstream communication platforms |
| E2 The basic knowledge is important | E122 You should understand how to train overall physical fitness |
| E3 Skills are also important | E123 Can design various exercise methods |
| E4 Know how to guide students when an emergency situation happens | E124 Can coordinate their spare time or time after work |
| E5 You should possess health communication relevant knowledge and skills | E125 It requires knowledge and ability to prevent some of the most basic and common diseases |
| E6 Has a relatively rich understanding of health education and health communication | E126 The knowledge of sports and health in books is a fundamental requirement for communication |
| E7 You need to understand the knowledge of relevant dissemination of communication and safety education | E127 Real experiences in teaching should be able to accumulate |
| E8 You need to understand the content of safety education | E128 Mastering some special health knowledge during learning |
| E9 Teacher should lead by example in their school | E129 It is necessary to have the ability to design sports rehabilitation activities during the recovering period |
| E10 When there are some high-risk actions, we can protect and guide students | E130 The ability to identify such potential health issues and provide timely communication and education is also important |
| E11 You should have relevant knowledge in this field | E131 Have the ability to collect plenty of information resources for application |
| E12 You need to have the ability to teach by example in this area | E132 You must possess professional knowledge |
| E13 You should set a good example for students | E133 Have the ability to use your professional knowledge via online communication |
| E14 Our words and actions will affect students | E134 Make sure that the health knowledge you spread is correct |

**Table A1.** Cont.

| **Tagged labels** | **Tagged labels** |
| --- | --- |
| E15 Can communicate by using the internet or even making an official account | E135 It requires some webcasting skills |
| E16 Have the abilities to identify the information which lacks effective supervision in new media platforms | E136 Can accept this change in character |
| E17 Should have a pure intention of helping people engage in healthy behavior | E137 You should have the ability to design communication contents for different age groups |
| E18 Should catch the fast train of epoch development | E138 Your communicate content should also be sufficiently specialized and targeted |
| E19 Should be able to grasp the peak period of short video platforms such as TikTok and Kwai | E139 You should also have the ability to differentiate different populations and professions when communicate |
| E20 Have the ability to apply information technology | E140 It requires ability to design and organize communicate scripts |
| E21 Has the ability to filter relevant information in the internet age | E141 You can use videos, PPT, or images to communicate |
| E22 You must be able to screen some things that are suitable for our communication objects to receive and learn | E142 You should be able to present these contents in PPT or similar software |
| E23 You should know the things to communicate to 10-year-old children and 15-year-old children are completely different | E143 Students who only use words to communicate may not fully understand |
| E24 It requires some understanding of computer operation | E144 Can make this kind of health knowledge related to sports into short videos |
| E25 You should be able to organize many activities in schools to spread health | E145 Can use different online platform to communicate |
| E26 Can establish some health-knowledge-related competitions | E146 It needs support from sport skills |
| E27 Can catch the special communication occasion, such as what to do after being injured | E147 It requires you to have the ability to integrate and integrate your communicate contents |
| E28 It requires the ability to organize sports and health competitions | E148 It requires you to be familiar with the content you are communicating |

**Table A1.** Cont.

| **Tagged labels** | **Tagged labels** |
| --- | --- |
| E29 Have innovative ability to convert and develop multiple functions for some devices | E149 Your communication content must be acceptable to others |
| E30 You should be able to design exams for health education | E150 Needs to be able to select useful content or resources on the internet to communicate |
| E31 Can set knowledge competitions related to health level evaluation | E151 It is necessary to clarify the concept of health first |
| E32 It requires teachers to have innovative abilities | E152 Understand the benefits of exercise on mental and physical health |
| E33 You should be able to communicate through multimedia | E153 You should have a high degree of health awareness |
| E34 The health communication does not necessarily require knowledge, it also requires skills | E154 You must have professional ability so that you will not be confused when doing it |
| E35 You must be good at health-related knowledge and skills | E155 There must be a spirit of contentment |
| E36 What you communicate must be correct | E156 You must have health-communication-related professional quality |
| E37 Need to be able to create a very good and eye-catching video for everyone to watch | E157 Know what benefits this sport can bring to people |
| E38 Know how to present the contents well to others in | E158 Be able to demonstrate the correct actions to your target audience |
| E39 It definitely requires the ability to edit | E159 You must be able to master the minimum use of multimedia equipment |
| E40 We need to be able to complete it with students through our own demonstration | E160 You must have teacher morality when communicating |
| E41 knows the methods for injury and timely hemostasis, as well as the different dressing methods for different parts | E161 It requires strong subjective initiative |
| E42 Health behaviors and awareness such as self-protection and other protection can be infiltrated in course teaching | E162 You must also be able to grasp the trend of young people |

**Table A1.** Cont.

| **Tagged labels** | **Tagged labels** |
| --- | --- |
| E43 It is necessary to be able to continuously improve oneself and ensure continuous and effective health guidance | E163 The ability to choose topics is very important |
| E44 Give others a bowl of water, and yourself have a bucket of water | E164 You must have some relatively objective judgments |
| E45 Master how to make short videos, such as cultivating the ability to edit videos and insert materials | E165 Your health literacy must reach a certain height |
| E46 When selecting topics, be able to carefully select according to the content and objects | E166 You must be able to pass health-related exams |
| E47 It requires PE teachers to have the ability to teach students in accordance with their aptitude | E167 The most basic dimensions of health professional knowledge need to be understood |
| E48 To be able to provide learning materials and health information suitable for students of different ages | E168 Once you have relevant sports and health knowledge, you must spread it |
| E49 You must have relevant knowledge | E169 Information literacy or digital literacy is important |
| E50 You need to know what kind of exercise can achieve what kind of effect | E170 New media and digital communication is very important now |
| E51 You must have interest and enthusiasm in this area | E171 Interpersonal communication ability is also very important in non-digital situations |
| E52 Have self-confidence and believe that physical fitness can be improved through exercise | E172 The ability to communicate is still very important |
| E53 Must have personal experience in this area | E173 You should be able to restructure your own health knowledge |
| E54 Must have the ability to enlarge the communication target | E174 Need to have the ability to restructure and frame the text |
| E55 Your communication must be systematic | E175 Needs to have the ability to generate text and tell stories |
| E56 Can impress others and make them believe | E176 You need to have the ability to process information |

**Table A1.** Cont.

| **Tagged labels** | **Tagged labels** |
| --- | --- |
| E57 Can teach them with relevant knowledge, including various exercise techniques | E177 Communication skills are also essential |
| E58 Have a thorough understanding of health communication | E178 Have a relatively scientific understanding of the content of communication |
| E59 Possess a certain level of planning ability | E179 Primary and middle school health education have different requirements at each level, and these seven aspects in national PE curriculum standards should be mastered |
| E60 It is not enough to have a little knowledge; it also requires systematic thinking | E180 The dissemination of health-related content is the main focus in the national PE curriculum standards |
| E61 Understand the various aspects included in health and the effects achieved through specific exercises | E181 The first thing to do is to meet the requirements outlined in the curriculum standards |
| E62 Understand that health communication is a systematic project | E182 Have relevant knowledge and understanding |
| E63 Have the ability to plan on your own | E183 Approach it from the perspective of changing their knowledge and belief attitudes |
| E64 Have a certain level of organizational ability | E184 Be able to communicate through current media and other means |
| E65 How to plan and organize your content is also important | E185 Can conduct scientific dissemination |
| E66 Have the ability to gradually develop professionalism | E186 Have a certain knowledge foundation |
| E67 The selection of the target audience for communication is crucial | E187 Have knowledge of preventing and responding to sports injuries |
| E68 The selection of communication channels is important | E188 Have good communication skills |
| E69 Communication must be approached from a more scientific perspective | E189 Effective communication is essential for communication |
| E70 Be able to differentiate the target audience for communication | E190 Mainly delivered through speaking or lecturing in the classroom |

**Table A1.** Cont.

| **Tagged labels** | **Tagged labels** |
| --- | --- |
| E71 Being able to disseminate with targeted approaches and methods | E191 You can only teach students how to use these devices if you understand how to use them yourself |
| E72 Being able to personally demonstrate and teach relevant massage techniques | E192 You need to be familiar with your own sports projects |
| E73 Being able to impart knowledge on sports health, sports injuries, and sports rehabilitation | E193 Having an awareness of sports injury prevention |
| E74 Having the ability to explain and demonstrate effectively | E194 Being able to deliberately incorporate this knowledge into the classroom |
| E75 Being able to express the technical essentials of movements clearly and accurately | E195 Having a sense of responsibility |
| E76 Having the ability to adapt and respond flexibly | E196 Being able to develop personalized plans |
| E77 Being able to seize special opportunities for dissemination | E197 Having some reserves of knowledge about health |
| E78 The content of dissemination should be more rigorous and the level of professionalism should be higher | E198 Knowing relevant knowledge about health in order to act on it |
| E79 One's own professional knowledge is the primary foundation of ability | E199 Needing to have a certain level of physical fitness |
| E80 Making others understand is essential | E200 Needing to possess the ability to demonstrate and showcase |
| E81 The organization and expression of language are crucial | E201 Language expression is crucial |
| E82 Proficiency in computer skills may indeed be important | E202 Personal motivation is also important |
| E83 The ability to create and manage documents is also necessary | E203 It requires your active efforts to exercise and cultivate students' willingness |
| E84 Having a certain courage and mindset | E204 You need to be proficient in using online dissemination tools |

**Table A1.** Cont.

| **Tagged labels** | **Tagged labels** |
| --- | --- |
| E85 Have the ability to dare to try | E205 Be able to use common social media platforms |
| E86 Enhance the effectiveness of dissemination by seizing special opportunities | E206 Have a deep understanding of the subject matter to be communicated |
| E87 Know which exercises can improve the health of the target audience | E207 Acquire a systematic understanding of sports and health-related topics |
| E88 Differentiate between male and female students when conducting dissemination | E208 Provide guidance in specific physical conditions |
| E89 Differentiate based on different venue designs and environmental conditions | E209 Possess targeted knowledge and understanding |
| E90 The importance of one's theoretical knowledge reserves | E210 Understand which projects are suitable for your communication |
| E91 Ensure a clear understanding of what is being disseminated | E211 Ensure that the content of health communication is scientifically grounded |
| E92 Have relevant experiences and engage in regular reflection | E212 Exercise quality control over the communicated content |
| E93 Transform and absorb cutting-edge theories and methods for dissemination | E213 Be able to guide students in preventing sports injuries in unexpected moments |
| E94 Convert practical experiences from teaching research into content for health dissemination | E214 Know how to perform specific exercises |
| E95 Understand the characteristics and qualities of students at each stage of learning | E215 Understand the fitness benefits behind each exercise |
| E96 Provide advice based on their own unique circumstances | E216 Differentiate the characteristics of different target groups |
| E97 Disseminated content should be practical and tailored | E217 Possess online communication capabilities |
| E98 Possess comprehensive abilities | E218 Have a decent level of proficiency in using information technology devices |
| E99 Understand the real needs of students in order to genuinely help them | E219 Be able to create rich and engaging health communication content |

**Table A1.** Cont.

| **Tagged labels** | **Tagged labels** |
| --- | --- |
| E100 Possess the ability to integrate theory with practice | E220 Understand the use of online communication methods |
| E101 Analyze the primary health needs at the societal or school level | E221 Keep up with the changing trends and mainstream of the digital era |
| E102 Have personal health awareness and healthy behaviors and habits | E222 Familiarize yourself with popular communication tools |
| E103 Have a certain understanding of exercise methods and knowledge | E223 Be able to select the appropriate communication platforms |
| E104 Expand beyond the school environment and have the ability to communicate and collaborate | E224 Possess the skills to use video editing tools for creating short videos |
| E105 Incorporate relevant branding into the community | E225 Have the ability to design communication content systematically |
| E106 Create interactive small groups | E226 Be able to develop diverse materials |
| E107 Establish a relatively comprehensive teaching system | E227 Fill the framework with varied and rich content |
| E108 Possess strong professional knowledge to enhance credibility | E228 Content selection is crucial |
| E109 Select simple and easy-to-learn content from a variety of materials | E229 Differentiate and understand the target audience clearly |
| E110 Know what popular and trending fitness activities are currently | E230 The ability to choose suitable topics is important |
| E111 Establish a well-structured and organized health system | E231 Know how to design activities or themes effectively |
| E112 Pay attention to the details and ensure the quality of the content | E232 Have a clear understanding of how to express the intended message |
| E113 Be able to record the 18-session module as videos and deliver them online | E233 Avoid blind actions and maintain a planned approach |
| E114 Have sufficient expertise and a sufficient knowledge base | E234 Ensure that the communication is based on a scientific foundation |

**Table A1.** Cont.

| **Tagged labels** | **Tagged labels** |
| --- | --- |
| E115 Have a certain level of research and understanding of the psychological aspects of sports for different age groups | E235 Be able to discern information found online |
| E116 Be able to cater to the specific needs of different target audiences | E236 Organize and categorize a wide range of materials effectively |
| E117 Understand the needs of different age groups, including younger and older individuals | E237 Possess a strong sense of responsibility |
| E118 Possess the ability to understand the psychology of the target audience | E238 Take action to implement your ideas |
| E119 Be skilled in computer technology and proficient in using various apps for live streaming | E239 Demonstrate perseverance and commitment |
| E120 Be able to flexibly utilize relevant apps for video recording, editing, and post-production | E240 Develop innovative and creative content |

**Table A2.** Conceptualization results of 240 tagged labels.

| **Number** | **Conceptualization concepts** | **Tagged labels** |
| --- | --- | --- |
| D1 | Having a healthy lifestyle | E1, E43, E53, E102 |
| D2 | Possessing relevant knowledge of sports and health | E2, E5, E6, E7, E8, E11, E35, E49, E58, E73, E79, E90, E91, E108, E114, E122, E125, E126, E128, E132, E167, E178, E179, E186, E187, E197, E198, E206, E207 |
| D3 | Having relevant communication skills | E3, E34, E54, E55, E97, E100, E103, E168, E177 |
| D4 | Leading by example | E12, E13, E14, E44, E84, E85, E156, E160, E181, E193, E195, E238 |
| D5 | Seizing special opportunities for health communication | E4, E10, E27, E76, E77, E86, E130, E194, E213 |
| D6 | Can use the internet for communication | E15, E18, E19, E24, E133, E135, E144, E204, E217, E220 |
| D7 | Being able to discern valid health information | E16, E21, E22, E134, E212, E235 |
| D8 | Having passion for health communication | E17, E51, E52, E155, E161, E202, E203, E239 |
| D9 | Being proficient in using technology such as computers | E20, E39, E45, E82, E119, E120, E169, E218, E224 |
| D10 | Recognizing the diverse needs of different groups | E23, E46, E47, E48, E88, E117, E118, E137 |
| D11 | Can organize various activities to promote and communicate health | E25, E26, E28, E64, E65 |
| D12 | Adapting and developing multiple functions when communicating | E29, E68, E141, E142, E182 |
| D13 | Can design relevant physical education and health assessment content | E30, E31, E59, E165, E166 |
| D14 | Can create different health communication themes | E32, E37, E123, E129, E230, E231 |
| D15 | Being able to demonstrate health practices | E40, E42, E44, E74, E143, E158, E191, E192, E199, E200, E214 |
| D16 | Possessing good language expression and organizational skills | E56, E75, E80, E81, E140, E172, E188, E189, E190, E201, E232 |
| D17 | Having the ability to plan systematically | E59, E60, E61, E62, E63, E98, E107, E111, E147, E124, E233 |
| D18 | Demonstrating professionalism when communicating | E66, E69, E78, E138, E155, E164, E183, E185, E211, E215, E234 |
| D19 | Can select target audiences for communication | E67, E70, E139, E229 |
| D20 | Can choose appropriate content | E83, E109, E131, E150, E148, E209, E210, E228 |
| D21 | Can select suitable sports activities | E87, E89, E110, E157 |
| D22 | Cultivating habits of reflection and analysis | E92, E93, E94, E127, E236 |
| D23 | Understanding the target audience for communication | E95, E96, E99, E101, E115, E116, E149, E216 |
| D24 | Having the ability to facilitate communication and collaboration | E104, E105, E106 |
| D25 | Possessing the ability to process and refine content | E112, E113, E173, E174, E175, E176, E219, E225, E226, E227, E240 |
| D26 | Being adaptable to different roles | E136, E151, E152, E237 |
| D27 | Can seize current hot topics | E121, E162, E163, E221, E222 |
| D28 | Can utilize new media formats for communication | E33, E37, E38, E145, E159, E170, E184, E205, E223 |
| D29 | Can design tailored approaches for specific target groups | E71, E96, E196, E208 |
| D30 | Acquiring skills in sports and health-related areas | E41, E50, E72, E146 |

**Table A3.** Results of the categorization of 30 concepts.

| **Subsidiary categories** | **Concepts** | **Total number of labels** |
| --- | --- | --- |
| C1  Sports and health knowledge reserve | D2 Possessing relevant knowledge of sports and health  D18 Demonstrating professionalism when communicating | 29  11 |
| C2  Health beliefs | D8 Having passion for health communication  D26 Being adaptable to different roles | 8  4 |
| C3  Health behaviors | D1 Having a healthy lifestyle  D4 Leading by example | 4  12 |
| C4  Health risk and crisis perception competence | D5 Seizing special opportunities for health communication  D27 Can seize current hot topics | 9  5 |
| C5  Communication audience perception competence | D10 Recognizing the diverse needs of different groups  D19 Can select target audiences for communication  D23 Understanding the target audience for communication | 8  4  8 |
| C6  Language expression competence | D16 Possessing good language expression and organizational skills  D24 Having the ability to facilitate communication and collaboration | 11  3 |
| C7  Organizational and design competence | D11 Can organize various activities to promote and communicate health  D12 Adapting and developing multiple functions when communicating  D13 Can design relevant physical education and health assessment content  D14 Can create different health communication themes  D17 Having the ability to plan systematically  D22 Cultivating habits of reflection and analysis  D29 Can design tailored approaches for specific target groups | 5  5  5  6  11  5  4 |
| C8  Utilization of new media tools competence | D6 Can use the internet for communication  D9 Being proficient in using technology such as computers  D28 Can utilize new media formats for communication | 10  9  9 |
| C9  Communication content selection and processing competence | D7 Being able to discern valid health information  D20 Can choose appropriate content  D21 Can select suitable sports activities  D25 Possessing the ability to process and refine content | 6  8  4  11 |
| C10  Professional skills | D3 Having relevant communication skills  D15 Being able to demonstrate health practices  D30 Acquiring skills in sports and health-related areas | 9  11  4 |
